# Complexities of Infective Endocarditis in a Young Patient With Hypertrophic Obstructive Cardiomyopathy: A Case of Dual Valve Involvement and Neurological Complications

**DOI:** 10.7759/cureus.57437

**Published:** 2024-04-02

**Authors:** Ekrem Yetiskul, Mohammad M Hussain, Danyal F Khan, Salman Khan, Jonathan Spagnola

**Affiliations:** 1 Internal Medicine, Staten Island University Hospital, Staten Island, USA; 2 Engineering, Stevens Institute of Technology, New York, USA; 3 Cardiology, Staten Island University Hospital, Staten Island, USA

**Keywords:** infectious disease, structural heart disease, cardiology, septic emboli, infective endocarditis, hypertrophic obstructive cardiomyopathy

## Abstract

Infective endocarditis (IE) is a life-threatening infection predominantly affecting the endocardium and heart valves, commonly seen in older patients and those with pre-existing cardiac conditions. Although rare in younger individuals with intact cardiac valves, certain structural heart diseases such as hypertrophic obstructive cardiomyopathy (HOCM) can increase the risk. We present a unique case of a 39-year-old female with a known history of HOCM, a condition characterized by abnormally thickened cardiac muscle primarily affecting the left ventricle. This patient was diagnosed with group B streptococcus infective endocarditis. Notably, this case was complicated by septic emboli to the brain. This case underscores the significant risk of IE in patients with HOCM, a demographic usually less susceptible to IE. It underscores the importance of early recognition and aggressive management of IE, especially in patients with structural heart diseases.

## Introduction

Infective endocarditis (IE) is an infection of the endocardium and heart valves that can lead to significant morbidity and mortality with rapid decline if not diagnosed and treated promptly. This disease more frequently affects males and elderly patients over the age of 65. The incidence of IE has been increasing over the years, particularly in patients with pre-existing cardiac conditions and those who have undergone invasive procedures [1]. The development of IE requires endocardial disruption and damage. A young patient with healthy native cardiac valves is usually resistant to the formation of IE. However, in the setting of structural heart disease, specifically high-pressure flow states, it can damage the endocardium over time, leading to an increased propensity to form vegetations [2]. Hypertrophic obstructive cardiomyopathy (HOCM) can be an inherited condition caused by mutations in genes that control the structure and function of the heart muscle, leading to abnormally thickened cardiac muscle. This hypertrophy typically affects the left ventricle and can precipitate a high-pressure flow state. This physiology can lead to a myriad of complications that have been found to be associated with IE [2].

This case report will discuss the devastating presentation of a young 39-year-old female patient with a known history of HOCM found to have group B streptococcus infective endocarditis with septic emboli to the brain.

## Case presentation

We present a case of a 39-year-old female with a past medical history of HOCM, atrial fibrillation, insulin-dependent diabetes mellitus, hypertension, and hyperlipidemia who presented to the emergency department for fever and altered mental status along with shortness of breath. Her cardiac examination revealed a loud diastolic murmur and signs of volume overload. She was diaphoretic, ill-appearing, and oriented only to person and place without any focal deficits. Examinations for Brudzinski and Kernig signs were negative, and there was no evidence of neck stiffness. The vitals on admission and pertinent laboratory values are listed in Table 1 and Table 2.

**Table 1:** Vital signs obtained during triage in the emergency department.

Vital sign	Value	Reference range
Temperature	102.2°F	97.9°F-100.4°F
Heart rate	92 beats per minute	60-100 beats per minute
Blood pressure	84/52 mmHg	90/60-120/80 mmHg
Oxygen saturation	99%	95%-100%

**Table 2:** Pertinent laboratory findings during evaluation in the emergency department.

Pertinent laboratory findings	Value	Reference range
White blood cell count	16 K/uL	4.80-10.80 K/uL
Glucose	489 mg/dL	70-99 mg/dL
Creatinine	1.4 mg/dL	0.7-1.5 mg/dL
Troponin	0.35 ng/mL	<0.01 ng/mL
Lactate	2.9 mmol/L	<2 mmol/L

**Figure 1:**
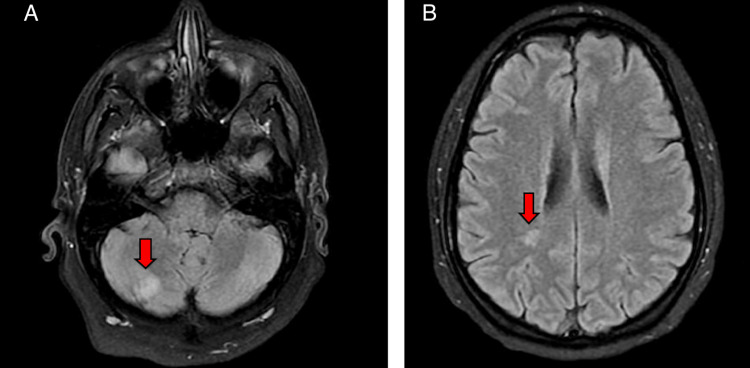
A and B demonstrate multiple acute infarcts throughout the supra- and infratentorial brain and brainstem noted on MRI of the head (red arrows). MRI: magnetic resonance imaging

**Figure 2:**
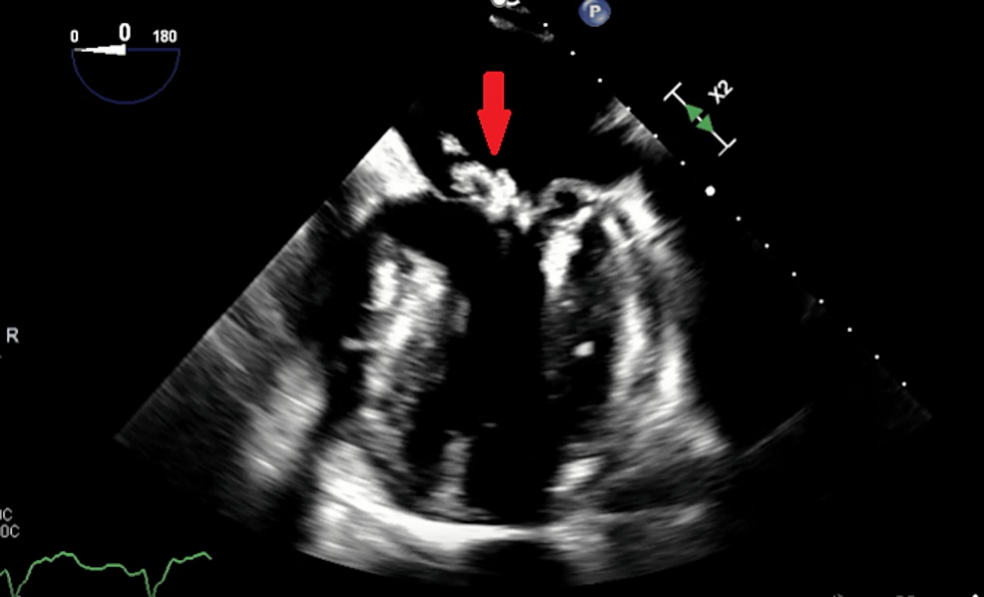
Mid-esophageal view with the red arrow pointing to a large mobile vegetation on the posterior mitral leaflet on TEE. TEE: transesophageal echocardiogram

**Figure 3:**
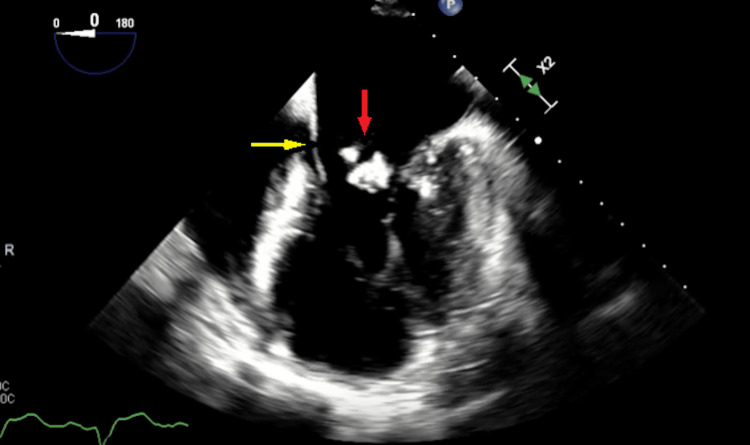
Mid-esophageal view with the red arrow pointing to a large mobile vegetation on the posterior mitral leaflet and the yellow arrow pointing to the anterior mitral valve leaflet.

**Figure 4:**
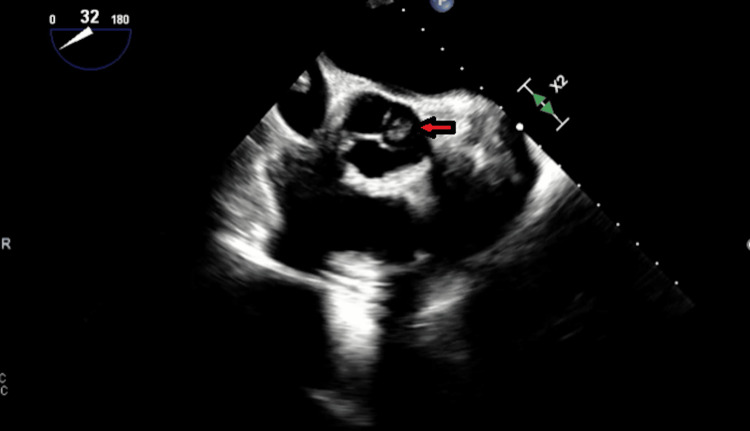
Mid-esophageal short-axis view of the aortic valve showing thickened aortic valve, and the red arrow is pointing toward a suspected vegetation.

**Figure 5:**
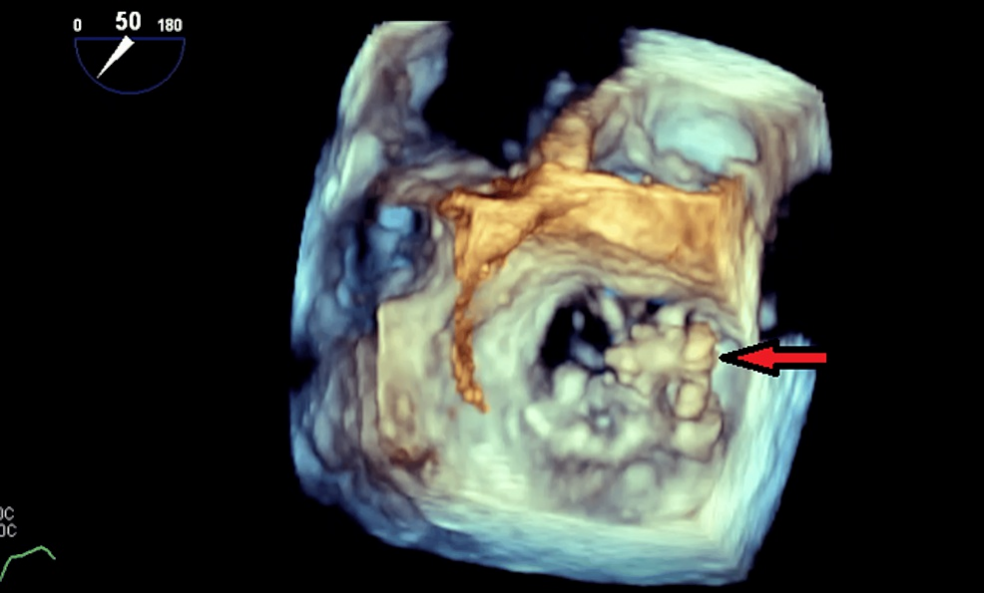
3D image of the mitral valve showing the surgeon's view with the red arrow pointing toward the posterior valve vegetation.

**Figure 6:**
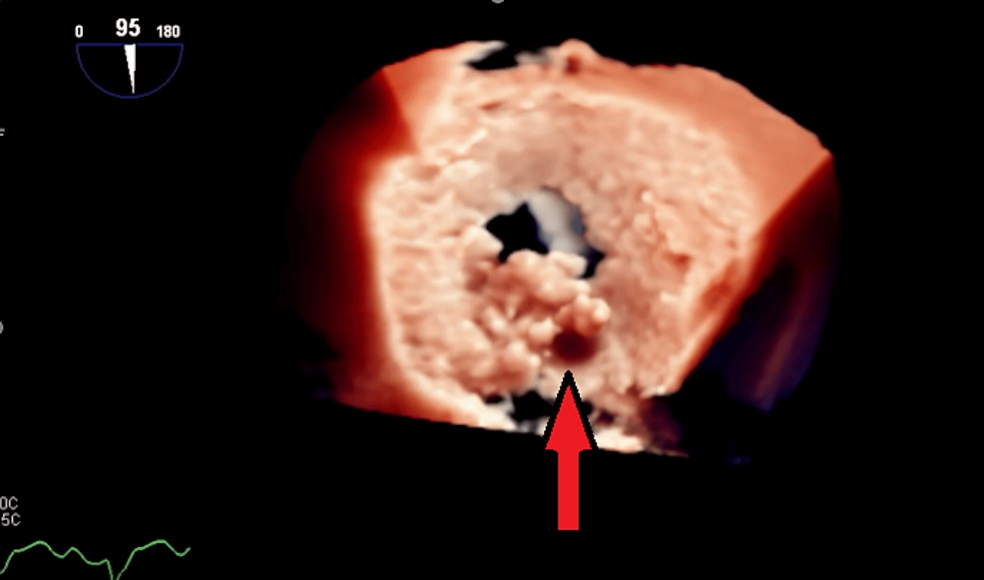
3D image of the mitral valve with the red arrow pointing toward the posterior valve vegetation.

The patient had a computed tomography (CT) of the head without contrast, which showed focal hypodensities involving bilateral cerebellar hemispheres, potentially reflecting age-indeterminate infarcts. These multivessel territorial infarcts were highly suspicious for septic emboli in the setting of septic shock. Blood cultures were drawn prior to the initiation of antibiotics. A lumbar puncture was attempted but was unsuccessful due to body habitus, after which the patient was started on empiric intravenous antibiotics with vancomycin and meropenem and goal-directed fluid resuscitation, followed by vasopressors for septic shock. The patient was also started on an insulin drip for diabetic ketoacidosis. The following day, the blood cultures revealed *Streptococcus agalactiae* (group B streptococcus), and antibiotic coverage was narrowed to ceftriaxone. An MRI of the brain was performed, which showed numerous acute infarcts throughout the supra- and infratentorial brain and brainstem (Figure 1). An echocardiogram was performed, which showed possible vegetations on the mitral and aortic valves. The valvular abnormalities were further investigated with a transesophageal echocardiogram (TEE) (Figures 2-6); significant findings included a large echogenic mobile mass on the posterior leaflet of the mitral valve consistent with vegetation with associated perforation of the leaflet, severe mitral valve regurgitation with a is centrally directed jet, systolic anterior motion of mitral valve and left ventricular outflow tract (LVOT) obstruction with a pressure gradient of 80 mmHg, thickening of the aortic valve suspicious of vegetation without evidence of peri-aortic abscess, and severe asymmetric left ventricular hypertrophy involving the septal wall (2.4 cm at the base). On day 4 of admission, the patient developed an acute focal neurological deficit in the right lower extremity (RLE) along with deficits in attention. A stroke code was activated, following which a CT angiography imaging revealed acute evolving infarcts highly suspicious for further embolization (Figure 3). The patient continued to worsen and aspirated, leading to acute hypoxemic respiratory failure and had to be intubated for airway protection.

**Figure 7:**
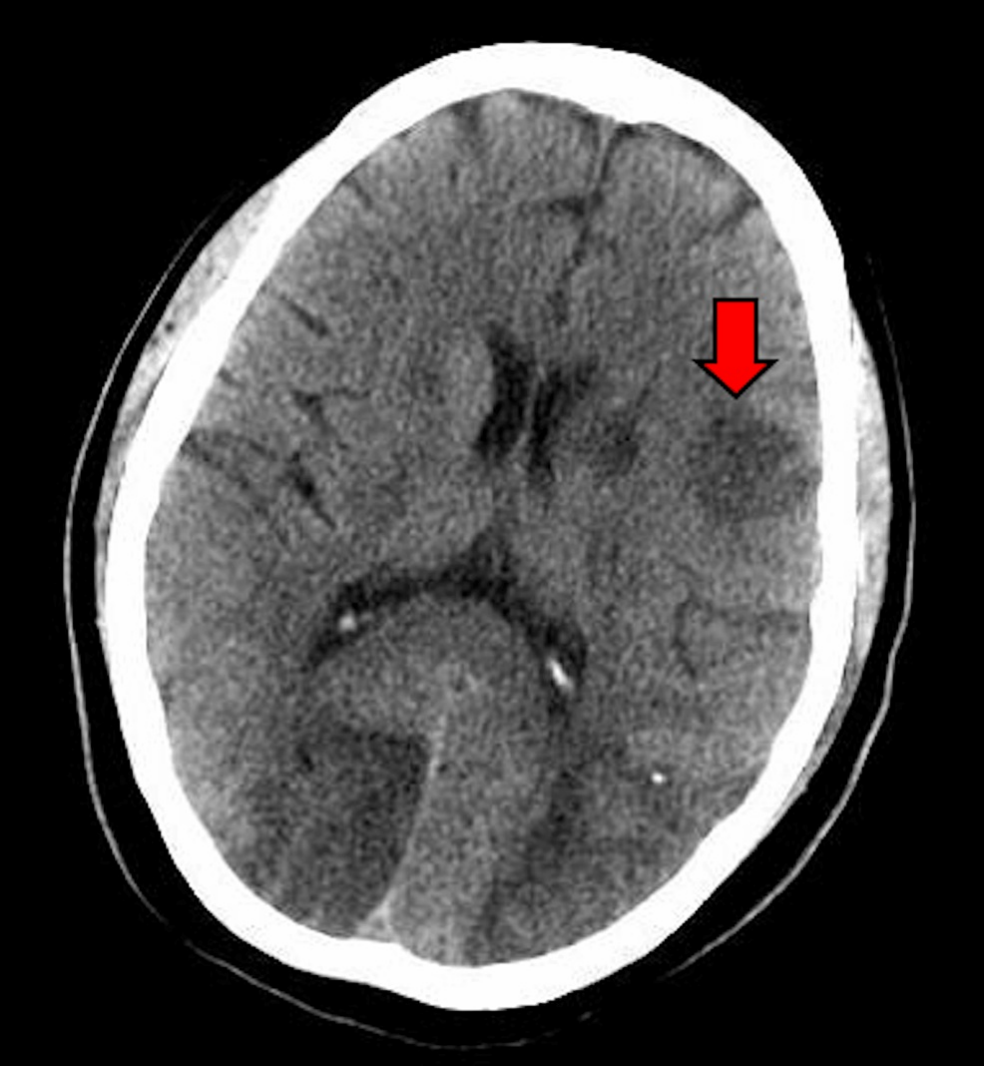
CT of the head demonstrating additional new infarcts during hospitalization (red arrow). CT: computed tomography

Cardiothoracic surgery evaluated the patient for surgical intervention, given embolic complications of infective endocarditis. However, the patient was deemed a poor surgical candidate, and the risk was deemed prohibitive at that time, given her acute stroke. A repeat CT of the head was performed on hospital day 7, which showed an extension of right occipital stroke (Figure 7). Neurology was consulted for neurological prognostication, and they deemed the patient to have a good potential for recovery despite the current stroke burden. A decision was made to get a second surgical opinion, and the patient was transferred to another nearby hospital, where she underwent successful mitral valve repair and was successfully weaned off the ventilator and discharged to a short-term rehabilitation facility.

## Discussion

Data on infective endocarditis in patients with HOCM is lacking and is mostly confined to case reports [3]. The incidence of IE in patients with HOCM is 1.4 per 1,000 person-years and is exclusive to patients with left ventricular outflow tract (LVOT) obstruction. The incidence increases to 9.8 per 1,000 person-years in patients with combined LVOT obstruction and left atrial dilation [2]. The most common valve involved is the mitral valve, particularly the ventricular aspect of the anterior mitral leaflet [3]. The postulated mechanisms of an increased risk of IE in HOCM patients include turbulent flow and eccentric mitral regurgitation leading to mitral valve damage [4]. To our knowledge, there is only one other case of posterior mitral valve leaflet IE with embolization in patients with HOCM [3]. Our case is the first reported case of IE involving both the posterior mitral leaflet and aortic valve with embolization in a patient with HOCM.

Duke's criteria for diagnosing infective endocarditis include major and minor criteria. The major criteria consist of positive blood cultures for infective endocarditis and evidence of endocardial involvement confirmed by echocardiogram [5]. Minor criteria include predisposition, such as a predisposing heart condition or intravenous drug use; fever; vascular phenomena such as major arterial emboli, septic pulmonary infarcts, or mycotic aneurysm; immunologic phenomena such as glomerulonephritis, Osler's nodes, Roth's spots, or rheumatoid factor; microbiological evidence not meeting the major criteria; and echocardiographic findings consistent with infective endocarditis, but not meeting the major criteria [5]. A diagnosis can be made when specific combinations of these criteria are met: either two major, one major and three minor, or five minor [5]. Antibiotics directed against the causative agent are the mainstay of treatment of IE in patients with concurrent HOCM; surgery should be considered when there are traditional indications such as abscess, significant hemodynamic compromise, or embolization [4]. The choice of vasopressors in patients with HOCM should be carefully tailored to the patient, considering echocardiographic and hemodynamic measurements. In patients with LVOT obstruction, phenylephrine has been shown to reduce the LVOT gradient [6]. Spirito et al. recommend antibiotic prophylaxis for preventing infective endocarditis solely in individuals with HOCM when accompanied by significant enlargement of the left atrium [2].

Medical therapy for patients with HOCM consists of B blockers, disopyramide, and calcium channel blockers to increase left ventricular (LV) filling and end-diastolic volume, leading to a reduction in the LVOT gradient [7]. Surgery for HOCM patients can also be considered if there is the presence of a resting or provocable LVOT gradient ≥30 mmHg at rest or ≥50 mmHg during exercise [7]. Nonsurgical septal reductive therapies can also be utilized in patients with HOCM and high LVOT gradients [8]. Early septal reduction therapy or mavacamten for patients with elevated LVOT gradients may possibly be explored to reduce the risk of IE from chronic mitral valve damage. Presently, data on the interventions discussed are unavailable, suggesting a possibility for further investigation.

## Conclusions

This case not only illuminates the intricate relationship between hypertrophic obstructive cardiomyopathy (HOCM) and the heightened risk of infective endocarditis (IE) but also emphasizes the criticality of early diagnosis and vigilant management in such patients. Patients with HOCM are particularly susceptible to IE due to valvular endothelial damage caused by abnormal hemodynamics. The presentation of IE in a 39-year-old female with HOCM, complicated by septic emboli to the brain, highlights the necessity for heightened clinical suspicion for IE in patients with HOCM presenting with signs of unexplained sepsis.
